# Effect of home-based rehabilitation of purposeful activity-based electrical stimulation therapy for chronic stroke survivors: a crossover randomized controlled trial

**DOI:** 10.3233/RNN-211157

**Published:** 2021-08-03

**Authors:** Seigo Minami, Yoshihiro Fukumoto, Ryuji Kobayashi, Hideaki Aoki, Tomoki Aoyama

**Affiliations:** a Department of Occupational Therapy, Osaka Kawasaki Rehabilitation University, Mizuma, Kaizuka City, Osaka, Japan; b Graduate School of Medicine, Kyoto University, Shogoinkawaracho, Sakyou-ku, Kyoto City, Kyoto Japan; c Department of Physical Medicine and Rehabilitation, Kansai Medical University, Shin-machi, Hirakata City, Osaka, Japan; d Graduate School of Human Health Sciences, Tokyo Metropolitan University, Higashi-Ogu, Arakawa City, Tokyo, Japan; e Graduate School of Medicine, Wakayama Medical University, Kimiidera, Wakayama City, Wakayama, Japan

## Abstract

**Background:**

In this trial we combined the effect of purposeful activity and electrical stimulation therapy (PA-EST) to promote transition of severely hemiparetic upper limb to auxiliary upper limb in chronic stroke survivors in a single-case study.

**Objective:**

The purpose of this study was to examine the effect of PA-EST on the upper limb motor function in a crossover randomized controlled trial.

**Methods:**

The study included eight stroke survivors (age: 63.1±10.9 years) who were receiving home-based visiting occupational therapy. The average time since stroke onset was 8.8±5.6 years. All participants had severely hemiparetic upper limb, with the Fugl–Meyer Assessment upper extremity (FMA-U) score of 21.3±8.5. Participants were randomly assigned to group A or B. Group A received PA-EST for 3 months (phase 1), followed by standard stretching and exercise for 3 months (phase 2), whereas group B had the inverse order of treatments. To avoid carry-over effect, 1-month washout period was provided between the phase 1 and 2. Two-way analysis of variance (ANOVA) with repeated measures was used for the analysis. The primary outcome was FMA-U, and the secondary outcomes were, Motor Activity Log (MAL; amount of use [AOU] and quality of movement [QOM]), and Goal attainment scale-light (GAS-light).

**Results:**

Repeated measures-ANOVA revealed a significant interaction between type of intervention and time for FMA-U (F = 16.303, P = 0.005), MAL AOU (F = 7.966, P = 0.026) and QOM (F = 6.408, P = 0.039), and GAS-light (F = 6.905, P = 0.034), where PA-EST was associated with significantly improved motor function and goal achievement compared with standard stretching.

**Conclusions:**

The PA-EST may have greater effects than stretch/exercise in the recovery of hand function as reflected in FMA-U, MAL, and GAS-light. Our results suggest that PA-EST is an important and useful home-based rehabilitation program for promoting the use of the severely hemiparetic upper limb in chronic stroke survivors.

## 1 Introduction

About 80% of stroke survivors experience paresis on one side of the body (Langhorne et al., 2009; Takashima et al., 2017). Patients with severe hemiparesis of the upper limb are often discharged to their homes without adequate in-hospital recovery of their hand function (Alexander, 1994). They have fewer opportunities to use their paretic hand during activities of daily living (ADL) at home, which may easily lead to the upper limb disuse and great reduction in their quality of life (Duncan et al., 2000; Lai et al., 2002). Therefore, effective treatment is required for patients with severe hemiparesis of the upper limb after hospital discharge.

Electrical stimulation therapy has been used as an effective treatment method for moderate upper-extremity hemiparesis (Alon et al., 2003; Mahmood et al., 2019; Sasaki et al., 2012). For example, Sasaki et al. (2012) examined the effect of 12-week functional electrical stimulation for the upper limb of stroke survivors with Brunnstrom recovery stage (BRS) III–IV, and found improvements in motor functions, range of joint motions, and cortical activations and alleviation of spasticity. Functional electrical stimulation has also been successfully used to treat the upper extremity in moderate chronic stroke survivors (Alon et al., 2003; The Japan Stroke Society, 2015). However, the effect of functional electrical stimulation for the severely hemiparetic upper limbs with BRS I–III (inability to move the wrist joint voluntarily) has not been analyzed.

We previously developed “purposeful activity-based electrical stimulation therapy (PA-EST)” program, which combines purposeful activities of occupational therapy and an electrical stimulation therapy device for rehabilitation of chronic stroke survivors with severely hemiparetic upper limbs (Minami et al., 2020). Our previous study (Minami et al., 2020) suggested that PA-EST could promote the use of a severely hemiparetic upper limb as an assistive hand via capturing the person’s thoughts and emotional patterns. However, it was only a case report and, therefore, insufficient to draw clinical suggestions.

Therefore, here we conducted a crossover randomized controlled trial with the aim to examine the potential benefits of home-based PA-EST program for chronic stroke survivors with severely hemiparetic upper limb compared with conventional stretching and exercises. We hypothesized that PA-EST would have a greater effect on motor function, use of paretic upper limb, and achievement of goals compared with the stretching/exercise training.

## 2 Materials and methods

### 2.1 Study design

The study design was a crossover randomized controlled trial. This study was conducted in accordance with the Declaration of Helsinki. It was approved by the Ethics Committee of Osaka Kawasaki Rehabilitation University (OKRU30-A018). The study was registered as a clinical trial in the University Hospital Medical Information Network (UMIN ID. 39663). This study was carried out from November 2018 to the end of November 2019.

### 2.2 Participants

We recruited community-dwelling chronic stroke survivors who were receiving home-based visiting occupational therapy. They were included after providing written informed consent. Preliminary tests (motor function test on the paretic side and cognitive function tests) were performed on candidate stroke survivors for screening against the inclusion and exclusion criteria. Inclusion criteria were as follows: (i) patients at least three years after stroke onset, (ii) patients with BRS I–III upper limb motor function, (iii) patients who could not extend their paretic wrist voluntarily toward dorsal for 10 degrees or more, (iv) patients receiving occupational therapy once per week, and (v) patients having sufficient cognitive functions to enable daily conversation. Exclusion criteria encompassed the following: (i) patients using a demand-type cardiac pacemaker, (ii) patients having a malignant tumor at the site of electrical stimulation, (iii) patients having fractures at the body site where electrical stimulation is applied, and (iv) patients whose physician did not permit them to receive electrical stimulation. In addition, the general health condition was targeted at those who were judged to be in good health. There were no restrictions on the type of stroke. There were no restrictions on taking medications to prevent secondary stroke.

All participants received home-based visiting occupational therapy at least once a week. The occupational therapy consisted of 40-min sessions that included range of motion training, strength training, and exercise involving occupational activities with the aim of maintaining and recovering daily life. The occupational therapy was continued during PA-EST or stretching/exercise intervention in this study.

### 2.3 Randomization and study schedule

For this crossover randomized controlled trial, the stroke survivors who matched the selection criteria were randomly assigned to group A or B using stratified block randomization technique. Participants were stratified by sex, age (<50 or≥50 years), and motor function of the paretic limb (Fugl–Meyer Assessment upper extremity [FMA-U]<30 or≥30 points). Then, randomization was conducted in each block using random numbers generated by Excel (Microsoft, Redmond, WA, USA). When a block of subjects was assembled, it was assigned to the group that would result in the smallest between-group difference in the initial FMA-U points.

The study schedule was follows: In group A, participants received PA-EST for 3 months as phase 1, and then received standard stretching and exercise for 3 months as phase 2. In group B, participants received standard stretching and exercise for 3 months as phase 1, followed by PA-EST for 3 months as phase 2. For both groups, 1-month washout period was provided between the phase 1 and 2 to avoid carry-over effect.

The occupational therapist confirmed manner of the PA-EST or stretching and exercise, and evaluated unpleasant subjective and objective symptoms during intervention and daily life, when a weekly home-based visiting occupational therapy was performed. When necessary, additional instructions for method of the PA-EST or stretching and exercise were provided by occupational therapist.

### 2.4 PA-EST

The electrical stimulator used in the PA-EST was an orthosis-type assisted functional electrical stimulator, NESSH200® (Bioness Inc., Valencia, CA, USA). This device has been classified as a medical device by the Japanese Ministry of Health, Labor, and Welfare. This electrical stimulator ensures quality, effectiveness, and safety of medical devices. As for the specifications, it can be a functional electrical stimulator that elicits complex movements by means of five pole electrodes. The five electrodes were arranged in the following muscles: extensor digitorum, extensor pollicis brevis, flexor digitorum superficialis, flexor pollicis longus, and thenar muscles. The electric stimulus was performed with the stimulation frequency of 36 Hz.

As the first step of PA-EST, the occupational therapist interviewed the participants to understand their subjective values, motives, and abilities accumulated through patients’ experiences as well as to grasp the participants’ thought and emotional patterns. This was based on the occupational behavior model, named as “the model of human occupational therapy” at the foundation of the practical theory of occupational therapy (Kielhofner & Forsyth, 2008). In PA-EST, each individual was assessed while being engaged in a voluntary activity, and the assessment focused on and respected the individual’s ADL and behavioral patterns, uniqueness, and their preferred way of performing tasks. More specifically, we assessed whether their environment could meet daily tasks and maintain ADL, and whether the individual could feel his(her) identity and execute daily life behavior.

The training components were selected specifically to focus on the severely hemiparetic upper limb (for example, for making a hamburger that her husband liked, holding hands with her grandchild, and opening the lid of coffee). For instance, for making a hamburger, electrical stimulation was applied to the paretic upper limb to energize the muscles while the participants actually performed a practice of mimicking a hamburger (Minami et al., 2020). All exercises were performed with the participants in the sitting or standing position. Adequate instructions and demonstrations were given by an occupational therapist on the first day of the intervention. During 3-month intervention period, participants performed unsupervised PA-EST at least 3 days per week, and an additional supervised PA-EST with the occupational therapist was applied at least once a week. The implementation time was 10–20 min per session, and participants were allowed to perform the PA-EST in two sessions per day.

### 2.5 Stretching and exercise

The training components were selected specifically to focus on the paretic side shoulder, elbow, and wrist. The exercise program included the following exercises: holding hands (i), shoulder lifting and lowing/stretching, (ii) elbow bending and extending/stretching, and (iii) wrist bending and extending/stretching. All exercises were done with the participants in a sitting position. Adequate instructions and demonstrations were provided by an occupational therapist on the first day of the intervention. During 3-month intervention period, each participant repeated the unsupervised stretching and exercise training at least 3 times per week. The implementation time was 10 to 20 min per session, and participants were allowed to perform the stretching and exercise in two sessions per day.

### 2.6 Assessment items

The outcomes of interest were measured 4 times, before and after the phase 1 and phase 2. The primary outcome was FMA-U (Bohannon & Smith, 1987; Gregson et al., 1999), and the secondary outcomes were Motor Activity Log (MAL; amount of use [AOU] and quality of movement [QOM]) (Taub et al., 1993; Uswatte at al., 2005; Van der Lee et al., 2004), Goal attainment scale-light (GAS-light) (Turner-Stokes, 2009; Turner-Strokes, 2020), and ultrasound-derived muscle thickness of upper limb and abdominal muscles. The measurements were performed by a single investigator for all participants. FMA is a measure of the overall physical function of stroke patients, and the items include motor function, range of motion, pain, sensation, and balance (Bohannon & Smith, 1987; Gregson et al., 1999). In this study, the FMA of motor function included the upper limbs (maximum score, 66 points). MAL is a measure of quantifying the patients’ subjective functional level for the usage of the paretic side during daily life. The MAL consists of 14 items. For each item, AOU and QOM of the paretic upper limb were evaluated on a scale from 0 to 5, with higher score indicating greater use and quality. Subsequently, an average of 14 items was obtained for AOU and QOM, separately. GAS-light is a method of scoring how well a patient’s individual goals are achieved during the intervention. Goals were set by the therapist and patients (e.g., ability to hold down ingredients by the paretic hand when cooking). GAS-light was scored from -2 to+2 points, with higher score indicating greater achievement. Muscle thickness (cm) of biceps brachii, brachioradialis, rectus abdominis, external oblique, internal oblique, and transversus abdominis was measured using ultrasound (LOGIQ e V2, GE Healthcare UK, Chalfont, Buckinghamshire, UK). Transducer was placed at the largest muscle diameter at the two-third of the upper arm length for biceps brachii, at proximal third of the forearm length, just lateral to the umbilicus for rectus abdominis, and at mid-point between the inferior rib and the iliac crest for abdominal oblique muscles.

### 2.7 Statistical analysis

Data were shown as the mean value±standard deviation (SD). Differences in age, time since stroke onset, BRS, and all outcome measures between the groups A and B at baseline were examined using unpared *t* test. A two-way repeated measures analysis of variance [type of intervention (PA-EST, stretching and exercise)×time (before and after intervention)] was performed to examine the effects of intervention. SPSS (ver. 26 IBM Japan, Inc.) was used as statistical processing software, and the significance level was set to 5% by a two-sided test.

## 3 Results

### 3.1 Participants

**Fig. 1:**
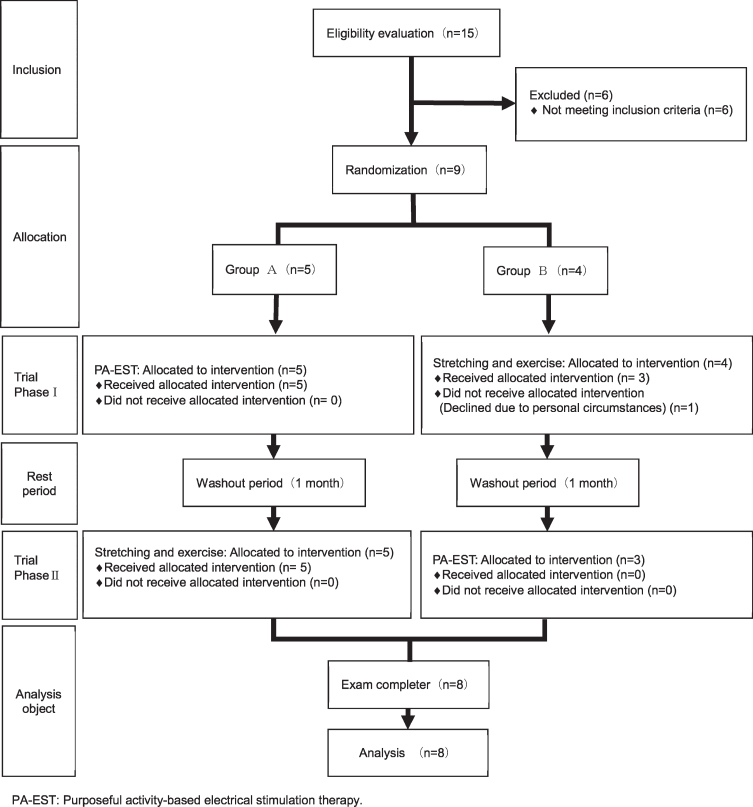
Flow chart of the study.

Figure 1 shows the study flow diagram. Fifteen chronic stroke survivors were recruited and screened. Of these, four participants aged over 80 years and two participants whose BRS was over III were excluded, and the remaining nine stroke survivors participated in this trial and were randomly assigned to group A (*n* = 5) and group B (*n* = 4). One participant from group B withdrew due to private reasons after phase 1. Therefore, a total of eight participants (five men, three women) who completed the phase 1 and 2 were analyzed. No participants experienced any adverse events during either intervention period. The mean age of the participants was 63.1±10.9 years, the mean time since stroke onset was 8.8±5.6 years, and the BRS was 2.8±0.5. At baseline, there were no significant differences in age and motor function between groups A and B (Table 1).

**Table 1:** Participants’ characteristics

Basic attributes	Overall		Group A		Group B		*P*-value
Sex (male : female)	5 : 3		3 : 2		2 : 1	
	Mean	SD	Mean	SD	Mean	SD
Attribute
Age (years)	63.1	10.9	64.0	13.4	61.7	10.4	0.734
Height (cm)	163.1	5.6	162.0	4.5	165.0	8.7	0.135
Body weight (kg)	65.1	13.0	63.4	5.5	60.0	8.2	0.734
BMI (kg/m^2^)	24.4	4.1	24.0	4.7	25.1	4.7	0.763
Pathology
Survival period	8.8	5.6	8.4	4.5	9.3	9.2	0.112
BRS (stage)	2.8	0.5	2.8	0.4	2.6	0.6	0.514
FMA-U (points)	21.3	8.7	21.2	9.7	21.3	8.5	0.778
MAL AOU	0.16	0.17	0.19	0.21	0.14	0.07	0.113
MAL QOM	0.14	0.13	0.16	0.16	0.19	0.08	0.169
GAS-light	29.1	1.8	29.0	2.2	29.1	1.6	0.728

SD: standard deviation; BMI: Body mass index; BRS: Brunnstrom recovery stage; FMA: Fugl–Meyer Assessment; FMA-U: FMA upper extremity; MAL: Motor activity log (AOU: amount of use; QOM: quality of movement); GAS-light: Goal attainment scale-light, Group A: Purposeful activity-based electrical stimulation therapy, group B: Stretching and exercise. In both group A and group B during both intervention periods, no participants experienced adverse events.

### 3.2 Difference in the effects of PA-EST and stretching/exercise training

The mean frequency of PA-EST was 4.25±1.83 days per week, and that of stretching and exercises was 4.00±1.69 days per week (P = 0.501).

FMA, MAL AOU and QOM, GAS-light, and muscle thickness before and after PA-EST and stretching/exercise are shown in Table 2. There was a significant interaction between the type of intervention and time for FMA-U (F = 16.303, P = 0.005), MAL AOU (F = 7.966, P = 0.026) and QOM (F =6.408, P = 0.039), and GAS-light (F = 6.905, P =0.034), with significantly improved motor function and goal achievement after PA-EST.

**Table 2:** Significant differences in the effects of PA-EST and stretching/exercise

Assessment item	PA-EST				Stretching/exercise				Type of intervention×time	
	Pre		Post		Pre		Post		*F*-value	*P*-value
	Mean	SD	Mean	SD	Mean	SD	Mean	SD
FMA-U	20.4	7.6	30.0	11.9	28.8	12.6	28.6	12.3	16.303	0.005^*^
MAL AOU	0.21	0.17	0.81	0.57	0.55	0.53	0.65	0.53	7.966	0.026^*^
MAL QOM	0.17	0.13	0.75	0.58	0.50	0.48	0.50	0.32	6.408	0.039^*^
GAS-light	32.1	5.2	49.5	8.3	43.4	13.5	49.3	12.5	6.905	0.034^*^
Brachioradialis	1.79	0.35	1.54	0.43	1.69	0.22	1.66	0.43	0.925	0.368
Biceps brachii	2.54	0.55	2.61	0.54	2.78	0.27	2.60	0.68	0.844	0.389
Rectus abdominis	0.91	0.19	0.92	0.22	0.92	0.24	0.92	0.26	0.007	0.937
External oblique	0.60	0.14	0.49	0.09	0.61	0.12	0.58	0.13	1.453	0.267
Internal oblique	0.94	0.26	0.89	0.30	0.93	0.27	0.86	0.21	0.048	0.833
Transversus abdominis	0.35	0.07	0.34	0.13	0.31	0.04	0.31	0.09	0.034	0.858

^*^P < 0.05. PA-EST: Purposeful activity-based electrical stimulation therapy; FMA: Fugl–Meyer Assessment; FMA-U: FMA upper extremity; MAL: Motor activity log (AOU: amount of use; QOM: quality of movement); GAS-light: Goal attainment scale-light.

Regarding muscle thickness, none of the measured muscles showed significant interaction between the type of intervention and time (brachioradialis: F = 0.925, P = 0.368; biceps brachii: F = 0.844, P = 0.389; rectus abdominis: F = 0.007, P = 0.937; external oblique: F = 1.453, P = 0.267; internal oblique: F = 0.048, P = 0.833; transverse abdominis: F = 0.034, P = 0.858).

## 4 Discussion

The purpose of this study was to evaluate the effects of PA-EST, the rehabilitation program combining purposeful activity and electrical stimulation therapy, for severely hemiparetic upper limb of chronic stroke survivors by assessing FMA, MAL, GAS-light, and ultrasound-measured muscle thickness. We hypothesized that PA-EST would have a greater effect on motor function, use of paretic upper limb, and achievement of goals, compared with the stretching/exercise training. The PA-EST had greater effects than stretch/exercise in the recovery of hand function as reflected in FMA-U, MAL, and GAS-light. Our results suggest that PA-EST is an important and useful home-based rehabilitation program for promoting the use of the severely hemiparetic upper limb in chronic stroke survivors. Thus, the hypothesis was mostly supported by our results.

Wolf et al. (1989) pointed out the risk that nonuse learned occurs in select neurological patients. In this study, PA-EST significantly improved motor function and use of the severely hemiparetic upper limbs compared with conventional stretching/exercise training. Moreover, based on the results of GAS-light, participants after PA-EST recognized that they achieved their goals better than participants after stretching and exercise. In this study, since there was no change in muscle thickness, it is unlikely that the improvement in motor function, AOU on the paretic side, and achievement of goals were caused by gain in muscle mass or strength. Sasaki et al. (2012) suggested that functional electrical stimulation has an effect on improving motor function measured using FMA and on cerebral cortex activation as measured by magnetic resonance imaging. Therefore, it is possible that the improvement in the motor function and the use of the paretic upper limb in our study resulted from an increase in activation of the cerebral cortex. The current study did not assess the cerebral cortex activity; thus, further study is needed to investigate the effect of PA-EST on cerebral cortex activity.

PA-EST was possible to perform with involvement of occupational therapists. After PA-EST sessions, FMA-U improved from 20.4 points to 30.0 points, and GAS-light score improved from 32.1 to 49.5. These results may indicate that the PA-EST is effective in converting a severely hemiparetic upper limb to the auxiliary upper limb in daily life.

Constraint-induced movement therapy (CIT) and repetitive facilitation exercises (RFEs) have been used as effective treatment methods for restoring the function of the paretic upper limb in patients with stroke and severe palsy. CIT is a treatment that increases the frequency of use of the paretic hand and upper limb via restraining or suppressing the unaffected side (Bonifer et al., 2005; Page & Levine, 2007; Taub et al., 1993; Taub et al., 2006; Uswatte at al., 2005). RFEs is a method that improves the affected upper limb’s function by repetitive facilitation methods (Kawahira et al., 2010). Although effective, those therapeutic approaches require the presence of a therapist, which is why it is difficult to apply them at home environment. In contrast, patients can do the PA-EST alone at home. Considering that stroke survivors are unable to undergo advanced rehabilitation adequately after discharge, leading to decline in their motor function, we suggest that PA-EST could be a useful unsupervised therapeutic method to enhance motor function and use of the severely paretic upper limb after hospital discharge.

In addition, it is essential to use the theory of occupational therapy to clarify the purposeful activity of PA-EST. Yerxa (1967) pointed out that occupational therapy helps the patient to recognize a task that enhances his/her presence by creating a strategy that allows the patient to perform a series of purposeful activities. Occupation is one’s own recognition of the values, motives, and abilities accumulated by the person’s experience in order to know the person’s thought and emotion patterns (Kielhofner & Forsyth, 2008). Mobily (1982) also pointed out that the body moves by increasing motivation. It is likely that PA-EST strengthened self-awareness of one’s will and ability by matching with the person’s purposeful activity, thus forming a certain order in life.

Our results suggest that PA-EST is an important and useful home-based program to promote using the severely hemiparetic upper limb as auxiliary upper limb in chronic stroke survivors. However, this study has some limitations. First, the stroke onset of the study participants was more than 3 years ago. Therefore, we cannot extrapolate the expectations to stroke survivors in whom stroke developed within 3 years. Second, the sample size was small, thus type 2 errors may have occurred in this study. Third, the washout period of this study was only 1 month; therefore, there is a possibility that the motor function and the degree of use of the paretic upper limb could not be washed out fully. In fact, the mean values of FMA-U, MAL AOU and QOM, and GAS-light before the stretching and exercise (see Table 2) were higher than those at baseline in the group B, which firstly performed the stretching and exercise (see Table 1). These findings may suggest that the washout period was not long enough to decline the effect of the PA-EST in group A, which firstly performed the PA-EST, and that a carry-over effect cannot be ruled out during 1 month. These results may also be associated with the fact that the number of participants in the group A (*n* = 5) was almost twice as high as that in the group B (*n* = 3), due to the small sample size in this study. Thus, many participants in this trial performed the stretching and exercise under condition that the effect of the PA-EST was carried over. Further large-scale studies with washout period longer than 1 month are needed to compare the net effect of the PA-EST and the stretching and exercise. Finally, this study did not measure functional changes in the brain. Therefore, it is unknown whether there was an activation of the cerebral cortex. Future studies should examine the functional changes of the brain by PA-EST with the transition of the chronically severely paretic upper extremity to the auxiliary upper extremity.

## 5 Conclusions

PA-EST, which combined purposeful activity and electrical stimulation therapy, may be more effective than stretching/exercise in motor function, use of paretic upper limbs, and achievement of goals in chronic stroke survivors with severe hemiparetic upper limb. Although further studies are needed to verify the findings, it is considered that PA-EST increased self-awareness of one’s will and ability by adjusting to joint movement and intentional activity by electrical stimulation.

## References

[ref001] Alexander, M. P. (1994). Stroke rehabilitation outcome a potential use of predictive variables to establish levels of care. Stroke, 25, 128–134. 8266360 10.1161/01.str.25.1.128

[ref002] Alon, G., Sunnerhagen, K. S., Geurts, A. C., & Ohry, A. (2003). A home-based, self-administered stimulation program to improve selected hand functions of chronic stroke. NeuroRehabilitation, 18(3), 215–225. 14530587

[ref003] Bohannon, R. W., & Smith, M. B. (1987). Interrater reliability of a modified Ashworth scale of muscle spasticity. Physical Therapy, 67, 206–207. 3809245 10.1093/ptj/67.2.206

[ref004] Bonifer, N. M., Anderson, K. M., & Arciniegas, D. B. (2005). Constraint-induced movement therapy after stroke: efficacy for patients with minimal upper-extremity motor ability. Archives of Physical Medicine and Rehabilitation, 86(9), 1867–1873. 16181956 10.1016/j.apmr.2005.04.002

[ref005] Duncan, P. W., Lai, S. M., & Keighley, J. (2000). Defining post-stroke recovery: implications for design and interpretation of drug trials. Neuropharmacology, 39, 835–841. 10699448 10.1016/s0028-3908(00)00003-4

[ref006] Gregson, J. M., Leathley, M., Moore, A. P., Sharma, A. K., Smith, T. L., & Watkins, C. L. (1999). Reliability of the Tone Assessment Scale and the modified Ashworth scale as clinical tools for assessing poststroke spasticity. Archives of Physical Medicine and Rehabilitation, 80, 1013–1016. 10489001 10.1016/s0003-9993(99)90053-9

[ref007] Kawahira, K., Shimodozono, M., Etoh, S., Kamada, K., Noma, T., & Tanaka, N. (2010). Effects of intensive repetition of a new facilitation technique on motor functional recovery of the hemiplegic upper limb and hand. Brain Injury, 24(10), 1202–1213. 20715890 10.3109/02699052.2010.506855 PMC2942772

[ref008] Kielhofner, G., & Forsyth, K. (2008). Model of human occupation: theory and application: therapeutic strategies for enabling change. 4th rev. edition. Lippincott Williams & Wilkins (Philadelphia, Pennsylvania, United States), 185-203.

[ref009] Lai, S. M., Studenski, S., Duncan, P. W., Duncan, P. W., & Perera, S. (2002). Persisting consequences of stroke measured by the Stroke Impact Scale. Stroke, 33, 1840–1844. 12105363 10.1161/01.str.0000019289.15440.f2

[ref010] Langhorne, P., Coupar F., & Pollock, A. (2009). Motor recovery after stroke: a systematic review. Lancet Neurology, 8, 741–754. 19608100 10.1016/S1474-4422(09)70150-4

[ref011] Mahmood, A., Veluswamy, S. K., Hombali, A., Mullick, A., Manikandan, N., & Solomon, J. M. (2019). Effect of transcutaneous electrical nerve stimulation on spasticity in adults with stroke: A systematic review and meta-analysis. Archives of Physical Medicine and Rehabilitation, 100(4), 751–768. 30452892 10.1016/j.apmr.2018.10.016

[ref012] Minami, S., Aoki, H., Kobayashi, R., Fukumoto, Y., & Aoyama, T. (2020). Transition of a severely hemiparetic upper limb to a supporting upper limb: Development of a purposeful activity–electrical stimulation therapy rehabilitation programme (A report of three cases. Journal of Japan Academy of Health Sciences, 23(1), 25–35.

[ref013] Mobily, K. E. (1982). Motivational aspects of exercise for the elderly: Barriers and solutions. Physical & Occupational Therapy in Geriatrics, 1, 43–53.

[ref014] Page, S. J., & Levine, P. (2007). Modified constraint-induced therapy in patients with chronic stroke exhibiting minimal movement ability in the affected arm. Physical Therapy, 87(7), 872–878. 17472950 10.2522/ptj.20060202

[ref015] Sasaki, K., Matsunaga, T., Tomite, T., Yoshikawa, T., & Shimada, Y. (2012). Effect of electrical stimulation therapy on upper extremity functional recovery and cerebral cortical changes in patients with chronic hemiplegia. Biomedical Research, 33(2), 89–96. 22572383 10.2220/biomedres.33.89

[ref016] Takashima, N., Arima, H., Kita. Y., Fujii, T., Miyamatsu, N., Komori, M., Sugimoto, Y., Nagata, S., Miura, K., & Nozaki, K. (2017). Incidence, management and short-term outcome of stroke in a general population of 1.4 million Japanese –Shiga stroke registry. Circulation Journal, 81, 1636–1646. 28579600 10.1253/circj.CJ-17-0177

[ref017] Taub, E., Miller, N. E., Novack, T. A., Cook, E. W. 3rd, Fleming, W.C., Nepomuceno, C. S., Connell, J. S., & Crago, J. E. (1993). Technique to improve chronic motor deficit after stroke. Archives of Physical Medicine and Rehabilitation, 74, 347–354. 8466415

[ref018] Taub, E., Uswatte, G., King, D. K., Morris, D., Crago, J. E., & Chatterjee, A. (2006). A placebo-controlled trial of constraint-induced movement therapy for upper extremity after stroke. Stroke, 37, 1045–1049. 16514097 10.1161/01.STR.0000206463.66461.97

[ref019] The Japan Stroke Society (2015). Japanese guidelines for the management of stroke 2015. Tokyo: Kyowa Kikaku Ltd.

[ref020] Turner-Stokes, L. (2009). Goal attainment scaling (GAS) in rehabilitation: A practical guide. Clinical Rehabilitation, 23, 362–370. 19179355 10.1177/0269215508101742

[ref021] Turner-Stokes L. (2020, February 22) Goal attainment scaling (GAS) in rehabilitation, the GAS-Light model. https://www.kcl.ac.uk/cicelysaunders/resources/tools/gas 10.1177/0269215508101742 19179355

[ref022] Uswatte, G., Taub, E., Morris, D., Vignolo, M., & McCulloch, K. (2005). Reliability and validity of the upper extremity Motor Activity Log-14 for measuring real-world arm use. Stroke, 36, 2493–2496. 16224078 10.1161/01.STR.0000185928.90848.2e

[ref023] Van der Lee, J. H., Beckerman, H., Knol, D. L., de Vet, H. C.W., & Bouter, L.M. (2004). Clinimetric properties of the Motor Activity Log for the assessment of arm use in hemiparetic patients. Stroke, 35, 1410–1414. 15087552 10.1161/01.STR.0000126900.24964.7e

[ref024] Wolf, S. L., Lecraw, D. E., Barton, L. A., & Jann, B. B. (1989). Forced use of hemiplegic upper extremities to reverse the effect of learned nonuse among chronic stroke and head-injured patients. Experimental Neurology, 104, 125–132. 2707361 10.1016/s0014-4886(89)80005-6

[ref025] Yerxa, E. (1967). Authentic occupational therapy. American Journal of Occupational Therapy, 21, 1–9. 6037915

